# Interleukin 1 blockade withcanakinumab for Hyper IGD syndrome (HIDS)

**DOI:** 10.1186/1546-0096-13-S1-P206

**Published:** 2015-09-28

**Authors:** J Brunnner, E Binder, D Karall, J Zschocke, C Fauth

**Affiliations:** 1 Medical University Innsbruck, Pediatrics, Innsbruck, Austria; 2 Medical University Innsbruck, Human Genetics, Innsbruck, Austria

## Introduction and question

Hyperimmunoglobulinemia D and periodic fever syndrome (HIDS; MIM# 260920) is a rare autosomal recessive autoinflammatory condition caused by mutations in the *MVK* gene, which encodes for mevalonate kinase. There is no standard treatment for HIDS. Therefore new therapeutic options might be developed.

## Methods and results of this case report

We report on a 2 year-old Austrian boy with recurrent episodes of fever, febrile seizures, arthralgias, and splenomegaly. Rash and abdominal pain were also seen occasionally. During attacks an acute-phase response was detected. Clinical and laboratory improvement was seen between attacks. These findings led to the tentative diagnosis of HIDS. Sequencing of the *MVK* gene showed a homozygous c.1129G>A (p.Val377Ile, also known as V377I) mutation in the child, while the healthy non-consanguineous parents were heterozygous. The mutation is known to be associated with HIDS.

Therapy with nonsteroidal anti-inflammatory drugs during attacks had poor benefit. A further febrile episode resulted in a status epilepticus. Treatment with canakinumab was initiated and a final dose of 4 mg/kg every 4 weeks resulted in the disappearance of febrile attacks and a considerable improvement of patient's quality of life during a 6-month follow-up period. The drug has been well tolerated, and no side effects were observed.

## Conclusion

Treatment with canakinumab is a therapeutical option for patients with HIDS.

## Consent to publish

Written informated consent for publication of their clinical details was obtained from the patient/parent/guardian/relative of the patient.

